# Mechanical mapping of the prostate in vivo using Dynamic Instrumented Palpation; towards an in vivo strategy for cancer assessment

**DOI:** 10.1177/09544119231154305

**Published:** 2023-04-16

**Authors:** Robert L Reuben, Steven J Hammer, Olufemi Johnson, Daniel W Good, Javier Palacio-Torralba, Antonio Candito, Yuhang Chen, Simon Phipps, Will Shu, S Alan McNeill

**Affiliations:** 1 Institute for Mechanical, Process and Energy Engineering, School of Engineering and Physical Sciences, Heriot Watt University, Edinburgh, UK; 2 Department of Urology, Western General Hospital, Edinburgh, UK; 3 Edinburgh Urological Cancer Group, Institute of Genetics and Molecular Medicine, University of Edinburgh, Edinburgh, UK

**Keywords:** Prostate cancer diagnosis, dynamic stiffness, relaxation time, elastic modulus, in vivo

## Abstract

A calibrated palpation sensor has been developed for making instrumented Digital Rectal Examinations (iDREs) with a view to assessing patients for prostate cancer. The instrument measures the dynamic stiffness of the palpable surface of the prostate, and has been trialled on 12 patients in vivo. The patients had been diagnosed with prostate cancer and were scheduled for radical prostatectomy. As far as possible, patients with asymmetric disease were chosen so as to give a variation in gland condition over the palpable surface. The device works by applying an oscillating pressure (force) to a flexible probe whose displacement into the tissue is also measured in order to yield a dynamic stiffness, the static stiffness being incidentally measured at the mean oscillatory force. The device was deployed mounted on the index finger of a urologist and measurements taken at 12–16 positions on each patient using light and firm pressure and palpation frequencies of 1 or 5 Hz. In parallel, conventional DRE assessments were made by a consultant urologist for cancer. After in vivo measurement, the glands were removed and examined histologically with each palpation point being classified as cancerous (C) or not (NC). The work has established the first measurements of static modulus of living prostate tissue to be: 26.8 (13.3) kPa for tissue affected by prostate cancer (C classification), and 24.8 kPa (11.9) for tissue unaffected by cancer (NC classification), values quoted as median (interquartile range). The dynamic properties were characterised by: dynamic modulus, 5.15 kPa (4.86) for the C classification and 4.61 kPa (3.08) for the NC classification and the time lag between force and displacement at 5 Hz palpation frequency, 0.0175 s (0.0078) for the C classification and 0.0186 s (0.0397) for the NC classification, values again quoted as median (interquartile range). With the limited set of features that could be generated, an Artificial Neural Network (ANN) classification yielded a sensitivity of 97%, negative predictive value of 86%, positive predictive value of 67% and accuracy of 70% but with relatively poor specificity (30%). Besides extending the feature set, there are a number of changes in probe design, probing strategy and in mechanics analysis, which are expected to improve the diagnostic capabilities of the method.

## Graphical abstract

**Graphical abstract:**
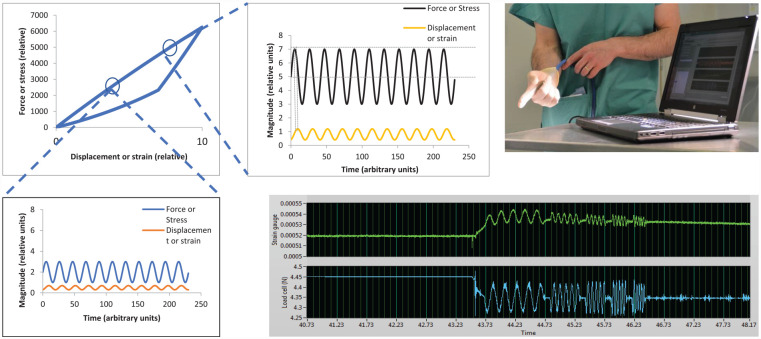

## Background

Currently, a blood test (for prostate specific antigen, PSA) and/or examination of the prostate by digital rectal examination (DRE) are used at the early stages of diagnosis of prostate cancer. DRE is used to assess tumour presence or absence over the palpable surface of the prostate, accessible trans-rectally. If age-referenced PSA results are used alone, negative biopsy rates can be as high as 75%,^1^ so, increasingly, biopsy is preceded by multi-parametric Magnetic Resonance Imaging (mpMRI). Those thought to be at risk for prostate cancer are assessed by biopsy, in which several core samples are removed from the prostate using a hollow needle, a process that may be guided by imaging techniques, such as trans-rectal ultrasound. The histological assessment of biopsy samples is a specialist matter, since the gland may be subject to benign disease and any cancer present may be diffuse. A system, known as the Gleason severity grade^2,3^ is currently used to classify prostate cancer on the basis of histopathological features, assessed by expert analysis of histological sections.

There is considerable interest in distinguishing between indolent and aggressive prostate cancers at the earliest possible stage to avoid unnecessary biopsies. There is no single test that can be used in primary care to differentiate unequivocally between prostate cancer that requires treatment and that which merely requires to be monitored, as DRE accuracy is poor and inter-rater variability is high.^4^ As well as being relatively expensive and requiring referral to secondary care, mpMRI also requires expert application and interpretation and there are some concerns about accuracy and inter-rater variability.^5^

Mechanical assessment of the prostate is also widely used in clinical practice in the form of elastography^6^ and its variants, in which sound waves are used to probe the structure of the soft tissue. Trans-rectal ultrasonography,^7^ trans-rectal sonoelastography^8^ and their combination with MRI continue to be evaluated in comparison with histopathology,^9^ as do new ultrasound modalities, such as the use of shear waves in conjunction with mechanical deformation of the prostate.^10^ Several commercially available clinical ultrasound scanners are available with these ultrasonic techniques although some types of prostate disease cannot be reliably distinguished due to stiffness artefacts.^11^

Quite recently, direct mechanical assessment of the prostate has attracted attention from both clinicians and engineers.^12^ Much of the interest is associated with robotic surgery (e.g. 13–15), but the principles are the same as those used here in that they seek to measure indentation stiffness by probing the surface of the prostate. A number of authors have developed point-probe devices and have measured prostate stiffness ex vivo to assess the potential sensitivity and specificity of tactile probing (e.g. 15, 16). With one exception,^17^ all of these studies treat the tissue as being elastic, with no time-dependent behaviour, which is only valid if measurements are made over very short or very long times relative to the relaxation time(s) of the tissue.^18^ Such an omission will inevitably lead to difficulties in interpretation of apparent elastic modulus as relaxation times are probably dominated by fluid movements in the deformed area of tissue. These relaxation times are expected to contain important diagnostic information as they will vary with the volume of material deformed as well as its local and global micro- and meso-scale morphology. To date, no direct mechanical measurements of prostate stiffness have been made in vivo, although one group^19^ have recently reported the use of a pressure probe deployed during robot-assisted surgery.

The current authors have pioneered a method^20^ to obtain elastic and viscous data on soft tissue, dynamic instrumented palpation (DIP). The method involves measuring dynamic stiffness using an oscillating indenter and its efficacy in discriminating between prostate cancer and other conditions has been demonstrated on tissue recovered from transurethral resections and cysto-prostatectomies.^21^

The instrument whose measurements are reported here was specifically configured for trans-rectal deployment, using dynamic instrumented palpation to generate complex modulus measurements over a range of frequencies. Because the probe is mounted on the clinician’s finger, it has been styled iDRE (instrumented DRE). This instrument has been trialled on 11 patients immediately before (in vivo) and shortly after (ex vivo) radical prostatectomy. The ex vivo results have already been published^22^ and, in this paper, results of DIP measurements in vivo prior to radical prostatectomy are presented using probes essentially the same as the one used for ex vivo measurement. The same patient group was used ex vivo and in vivo so that the target histological section data is the same, although the registration of probe point against histological sections is less precise and the control of the pre-strain is limited in vivo. However, the key difference is that the tissue being probed is alive and perfused, so these represent the world’s first measurements of prostate stiffness where the fluid component is physiologically connected.

## Methods

### Patient cohort

A full Regional Ethics Committee review and favourable opinion was granted prior to the commencement of the study, Ref: REC (12/SS/1048). Patients with prostate cancer who had elected to undergo surgery (laparoscopic radical prostatectomy; removal of the whole prostate and seminal vesicles) and had not had any previous treatment for prostate cancer were eligible for inclusion. Those where the diagnosis using DRE, mpMRI and Trans-rectal Sonoelastography (TRSE) indicated that a contrast could be expected between the two lobes were invited to participate and the 12 patients whose data is presented here gave their informed consent. Immediately prior to surgery, while the patients were anaesthetised, detailed conventional DRE assessment was carried on 12–16 zones (depending on the size of the prostate) for clinical T stage assessment and this was followed by the iDRE measurements. Each excised whole prostate was later sectioned and assessed for cancer in the area below each probe point as described elsewhere.^22^

Unregistered data on a single patient from a later study involving (to date) around 450 patients is also reported briefly here as it uses a further modified probe and an efficient protocol designed to minimise intervention time. In this case, the patient was undergoing a robot-assisted radical prostatectomy and the iDRE was carried out with the patient not anaesthetised prior to surgery.

### Instrumented DRE (iDRE) measurements

**Figure 1.**
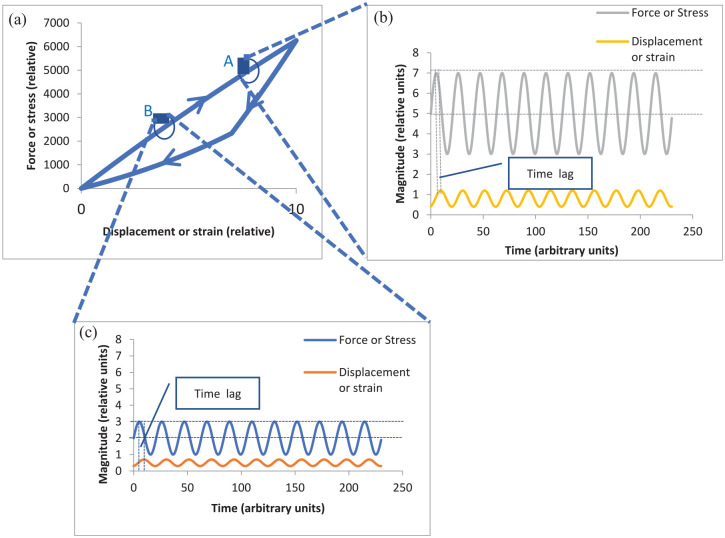
Illustration of the essential principles of DIP: (a) Load extension or stress-strain curve for loading and unloading at a given strain rate, (b) Oscillating force and displacement response at point A on load-displacement curve, (c) Oscillating force and displacement response at point B on load-displacement curve. (a): Loading curve for a given strain rate. (b): Response to an oscillating load of mean 5 units and amplitude 2 units. (c): Response to an oscillating load of mean 2 units and amplitude 1 unit.

Dynamic instrumented palpation (DIP) involves the measurement of the dynamic stiffness (or dynamic modulus) of a material by applying an oscillating force (or stress) and measuring the resulting displacement (or strain) or vice versa, the two modes being referred to as load control and displacement control, respectively. The dynamic response of a material reveals its elastic and its viscous behaviour and, in principle, the elastic and viscous response can vary with frequency, depth of modulation and pre-strain. The essential experimental variables in DIP are the frequency of oscillation, the amplitude of the applied force or displacement and the mean value of force or displacement around which oscillation is applied. Figure 1 illustrates the method schematically for a material which displays mechanical hysteresis, a phenomenon which can be described by a force-displacement behaviour which is different for loading and unloading 1(a). It should be noted that the hysteresis curve will be different for different loading rates and the loading curve need not be linear, so that the slopes at A and B could be different, even for a given loading rate. Figure 1(b) and (c) show schematic displacement responses for a fixed frequency of palpation force with two different mean values and two different amplitudes. For a homogeneous soft material, where the slope of the force-displacement curve increases with strain, it would be expected that a fixed amplitude and frequency of force will give rise to a reduced amplitude of displacement. However, the principle of the method is to search for modulus inhomogeneities (which may be below the surface) and the purpose of Figure 1 is to illustrate that static and dynamic stiffness can be used as a method of searching for cancer foci, where the parameters of pre-strain, force amplitude and frequency of force oscillation can be varied to optimise the visibility of tumours.

For a given set of palpation parameters (mean value, palpation amplitude and palpation frequency), three measures can be obtained: the static stiffness (mean force/mean displacement), the dynamic stiffness (amplitude of force/amplitude of displacement) and phase difference between the displacement and the force. It should be noted that, even for a fixed time-difference, the phase difference will vary with frequency of palpation, so that non-linear changes with frequency can reveal information about characteristic times associated with the tissue.

**Figure 2.**
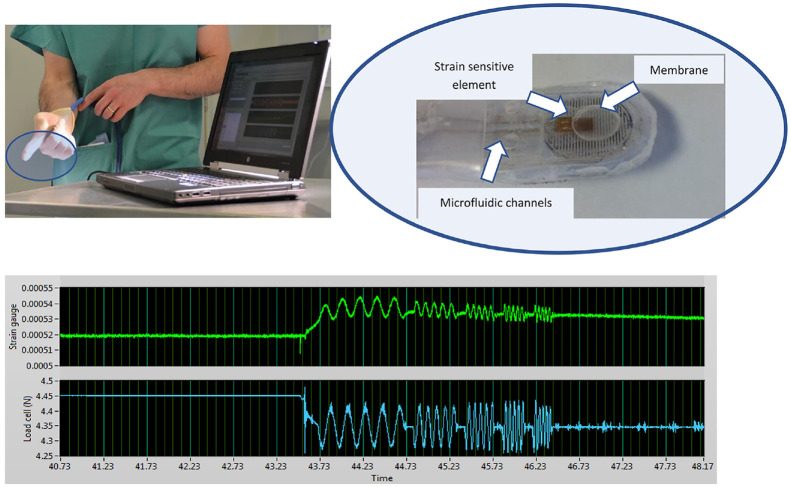
In vivo measurement arrangement. Top left: Probe mounted under clinician’s glove with data acquisition computer in foreground. Top right: Probe elements (photo from Hammer et al.^22^). Bottom: Typical (uncalibrated) load and displacement measurements for a single probe point with actuation frequencies of 5, 10, 15, 20 and 25 Hz.

The prototype in vivo device used here was of the same construction as reported earlier,^22^ with a flexible membrane being pressurised hydraulically and inflated against the prostate, the pressure being modulated at a nominally fixed amplitude about a fixed mean measured by a load cell within the control unit. The displacement of the probe into the tissue was measured by a strain gauge mounted directly onto the membrane. Only one probe was used for the in vivo studies and it was individually calibrated against a test machine using a series of phantoms of appropriate static stiffness. Figure 2 illustrates the arrangement used for in vivo testing, with the probe mounted under the clinician’s glove and the inset shows schematically the hydraulic channels, the membrane and the strain-sensitive element. It should be noted that the glove is relatively loose and serves principally as a prophylactic layer. Figure 2 also shows a typical uncalibrated output for the full protocol of five actuation frequencies; as can be seen the measurement time for a single probe point is only a few seconds. For best compatibility with the ex vivo measurements,^22^ only two frequencies (1 and 5 Hz) are reported here.

Two further points are worthy of note here. The first is that the approach is fundamentally different from rolling contact probing, in which the reaction force is measured as the probe is scanned over the palpable surface of the prostate.^15^ The discrete approach used here allows the viscous response of the tissue to be separated from the elastic response, potentially providing additional diagnostic information. The second point is that there is no a priori reason why there should be only one characteristic time for the tissue; in fact, the strain trace in Figure 2 shows a transient on first application of the actuator. In, principle, this information can also be extracted and used, although, at this stage, only the sinusoidal response once transients in the mean have been extracted is analysed and reported.

**Figure 3.**
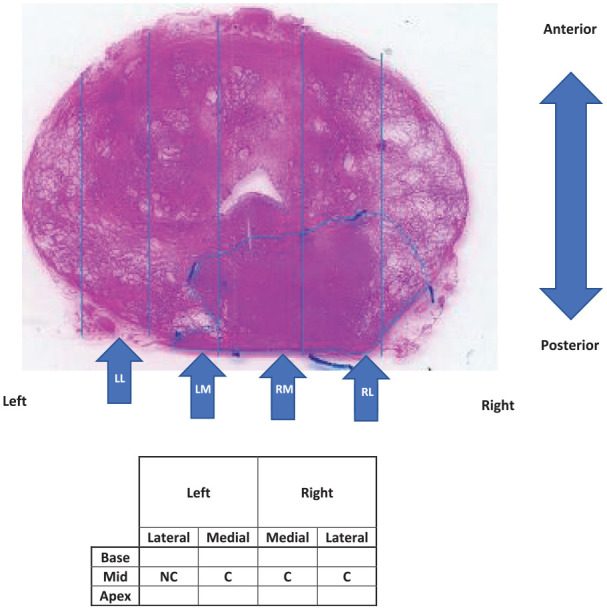
Process of classifying in vivo probe points as containing cancer (C) or not (NC). Shown is superior transverse mid-section of excised prostate with approximate positions of the four probe positions on the palpable surface in vivo.

Of the 12 patients (numbered P11-P22) assessed in vivo, complete data were obtained for 8 (P15-P22) at an actuation frequency of 5 Hz, and for 4 (P11-P14) at an actuation frequency of 1 Hz. The iDRE measurements were made at (typically) 12 points covering the palpable (posterior) surface (Figure 3) using both ‘light’ and ‘firm’ pressure, subjectively assessed by the clinician. Over the 12 patients, a total of 165 points were probed The in vivo tests were carried out immediately before radical prostatectomy and followed a conventional DRE using a protocol similar to that shown in Figure 3. The clinician will first find the base and move from left to right before moving down to the mid-section and then the apex. Depending on the patient, it may not be possible to obtain 12 points and/or these may overlap.

Once removed, each prostate was probed ex vivo at up to 35 locations^22^ covering the whole posterior surface, the position being marked using surgical clips which were retained in place until sectioning. Each point was classified on the basis of the histological section (Figure 3) as containing cancer (C) or not (NC). At this stage, no distinction in the target is made for the size or depth of any tumour, or the Gleason Grade of the cancer, and benign disease (such as BPH) was classified as NC.

Clearly, the registration of probe point with the histological sections was more precise for the ex vivo tests than the in vivo, but palpable lesions tend to be relatively large and the overlap of probe points on both the in vivo and ex vivo measurements makes this less of a concern, although it does add some imprecision to a point-by-point correlation (in both cases).

### Data conditioning and feature extraction

**Figure 4.**
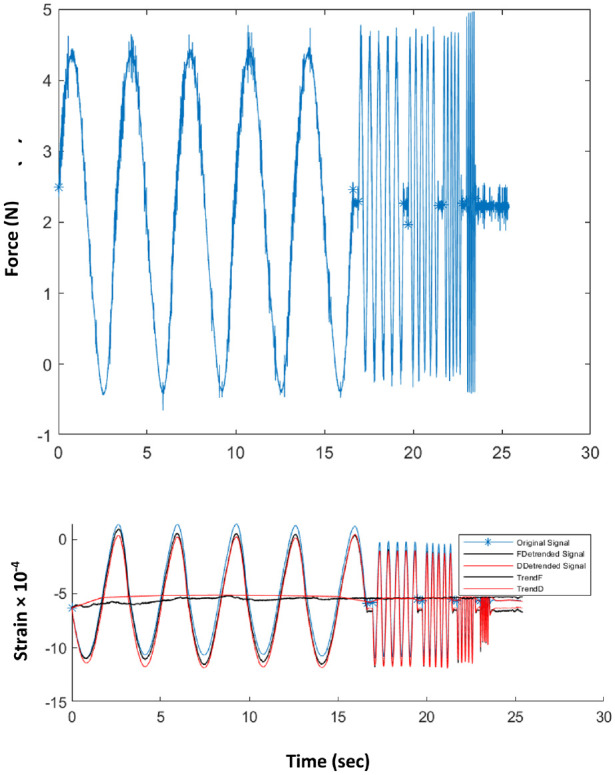
Example of recorded raw in vivo data at a given point for frequencies of 0.3, 2, 4, 5 and 10 Hz. Top: Force recorded at load cell on actuator output. Bottom: Strain recorded on strain sensitive element on diaphragm. Blue curve: as recorded; Black curves: trend of the average strain over each frequency and resulting de-trended strain signal; Red curves: trend of the average strain over all frequencies and resultingde-trended strain signal.

Figure 4 shows an example of raw patient data captured at a typical probe point. The force at the actuator is controlled and the response (indentation displacement) is measured on the strain sensitive element mounted on the membrane. Data were captured at a sampling rate of 1000 Hz over around 20 s and the signals averaged to produce a mean value, a mean amplitude and a phase at the fundamental actuation frequency for each of the strain and force. Prior to signal averaging, long-time transients are removed from the strain signal by subtracting a multi-point average from each individual strain measurement as shown in Figure 4. Using the calibration process described earlier,^22^ three characteristics of the strain gauge and actuator signals: the mean ratio (MR), amplitude ratio (AR) and the phase difference (PD) were calculated.

Mean ratio was calculated using:

(1) $$\mathrm{MR}=K_{f}\frac{\bar{F}}{\bar{\varepsilon }}$$

where $\bar{F}$ is the mean force measured at the actuator, $\bar{\varepsilon }$ is the mean strain and *K_f_* is a calibration factor that relates the actuator force to that at the proximal end and relates the (negative) strain to the displacement of the membrane into the tissue. The mean ratio thus gives a measure of the quasi-static stiffness of the prostate tissue at that point.

Amplitude ratio was calculated using

(2) $$\mathrm{AR}=K_{f}\frac{\hat{F}}{\hat{\varepsilon }}\;$$

where $\hat{F}$ is the amplitude of the actuator force, and $\hat{\varepsilon }$ is the amplitude of the strain, so that, AR gives a measure of the magnitude of the dynamic stiffness.

The calibration factors in equations (1) and (2) were derived from tests on gelatine using a conventional test machine.^22^

The phase difference between actuator force and strain completes the description of the dynamic response. The contact force (and hence depth of indentation before the membrane is oscillated) does not figure in the calculation of the stiffness map, although it needs to be recorded since both the dynamic and static stiffness can, especially in soft materials, vary significantly with mean stress or strain (see Figure 1).

Nominal values for static and dynamic elastic modulus, *E*, were calculated from the (calibrated) stiffness measurements, assuming a simple Hertzian contact model.^22^ At each probe point, the calibrated values (in kPa) of quasi-static elastic modulus (*E_static_*), dynamic modulus (*E_dynamic_*) and the tangent of the phase difference were calculated from the raw data by extracting the first Fourier components of the periodic responses after removing any trend in mean value (Figure 4). The phase differences were converted to time lags, *Δt*, using the fact that the periodic time is known and the recorded displacement signals are close to sinusoidal with a strong fundamental at the actuation frequency.

One of the objectives of the paper was to determine values of the mechanical properties of prostate tissue as it behaves in vivo. In order to compare these values between tissue classifications, between in vivo and ex vivo experiments and with other investigators, a simple statistical indicator was used. For each group of data, the median value and the upper and lower quartiles were calculated using a standard Excel function and results cited as median and interquartile range.

Another objective of the work was to assess the potential diagnostic capability of the mechanical features when considered as an assemblage. Because of the complexity of the mechanical information (six features at two contact pressures) and the heterogeneity of the column of tissue under the palpation point, even using the simplified classification, a pattern-recognition tool was used to assess in-patient sensitivity (i.e. proportion of points with disease which return a positive result from the mechanical indicators) and specificity (i.e. proportion of points without disease which return a negative result from the mechanical indicators). A proprietary algorithm (MatLab) consisting of a two-layer feed-forward Artificial Neural Network (ANN) was used, incorporating sigmoid neurons in the hidden layer and ‘softmax’ neurons in the output layer. The mechanical data at each point were input as a feature vector and the target consisted of a two- or four-element vector with zeroes in all but the correct classification (Table 1). In each ANN run, 10 iterations of training (using scaled conjugate back-propagation) were used, each time with the data set being divided randomly into three parts, 70% for training, 15% for validation and 15% for testing. This was chosen to give a reasonable compromise between training and stability and allowed comparison between the various choices of feature vector and data set described below.

**Table 1.** Statistical indicators of quasi-static modulus measured in vivo. Values quoted as median (interquartile range).

Patients	Pressure	Freq (Hz)	*n_C_*	*n_NC_*	*E_static_* C (kPa)	*E_static_* NC (kPa)
11–14	F	1	36	9	28.1 (19.8)	26.6 (8.9)
11–14	L	1	36	9	26.4 (8.1)	24.0 (1.7)
15–22	F	5	63	57	25.4 (13.8)	24.3 (12.2)
15–22	L	5	64	58	24.6 (17.2)	23.3 (9.2)
11–22	F	1 and 5	99	66	26.8 (13.3)	24.8 (11.9)
11–22	L	1 and 5	100	67	25.2 (12.8)	23.6 (6.4)

F: firm pressure applied; L: light pressure applied; C: point assessed as cancer; NC: point assessed as not cancer.

## Results and analysis

Of the 12 patients (numbered P11-P22) assessed in vivo with a single probe exemplar, 11 were also assessed in the programme of ex vivo testing reported earlier^22^ using multiple overlapping points for probing. This gives an opportunity to compare the in vivo and ex vivo results. Besides the fact that a different probe was used, the principal difference between in vivo and ex vivo testing is that the depth of pre-indentation could not be controlled and so the three depths of 3, 5 and 8 mm used in the ex vivo tests were replaced by ‘light’ and ‘firm’ pressure, subjectively assessed by the clinician.

Table 1 summarises the statistical indicators for quasi-static elastic modulus for various subsets of the data separated by finger pressure and actuation frequency. The numbers of NC and C points in each of the subsets (*n_NC_* and *n_C_*) are given, along with the corresponding median values of *E_static_*. The interquartile range is given in brackets after each value as a measure of spread of the measurement. As can be seen, there is a small, but consistent, increase in static modulus for those point measurements classified as C, although the spread of the data is too large for this measure to be used alone for identification of cancer point-by-point. Also, for all groupings of the data, firm contact pressure gives rise to larger measured values of static modulus, whereas the higher frequency gives consistently larger measured values of static modulus for both contact pressures and for C and NC classifications. Mixing the 1 and 5 Hz data does not eradicate the above observations about C versus NC and slightly ameliorates the interquartile ranges.

Table 2 summarises the statistical indicators for *E_static_* for the ex vivo measurements for the full patient set for both frequencies and disaggregated for each of the pre-strain depths. Although these data have been reported earlier,^22^ they are reported again here with a modified statistical indicator to make them compatible with Table 1. The calibrated values of modulus recorded ex vivo are about half those recorded in vivo and there is also very little difference between C and NC points. Although the ex vivo differences between C and NC are very small relative to the interquartile range, there is a reasonably consistent increase of a similar type to the in vivo measurements, and increasing pre-strain increases the values of recorded modulus and enhances the difference between C and NC. It seems likely that the stark differences between two types of measurement are due to a combination of the perfusion of the in vivo prostate, the different support conditions and, perhaps most significantly, the use of load control (albeit subjective) in vivo for the pre-strain rather than displacement control in the ex vivo tests.

**Table 2.** Statistical indicators of quasi-static modulus measured ex vivo. Values quoted as median (interquartile range).

Patients	Depth (mm)	Freq (Hz)	*n_C_*	*n_NC_*	*E_static_* C (kPa)	*E_static_* NC (kPa)
16–30	All	1 and 5	1260	898	14.9 (0.75)	14.8 (0.77)
16–30	3	1 and 5	420	298	14.7 (0.78)	14.8 (0.78)
16–30	5	1 and 5	420	300	14.9 (0.70)	14.7 (0.78)
16–30	8	1 and 5	420	300	15.0 (0.87)	14.9 (0.85)

F: firm pressure applied; L: light pressure applied; C: point assessed as cancer; NC: point assessed as not cancer.

Table 3 shows the statistical indicators for both the in vivo and ex vivo measurements for dynamic modulus. Since dynamic modulus can, in principle, vary with frequency, the 5 Hz in vivo and ex vivo data were used for the same patients in order to give congruency in the data sets. By complete contrast with the static modulus, the ex vivo measurements of dynamic modulus are substantially higher than in vivo although the difference between median values for C and NC is a little larger in vivo (about 10%) than ex vivo (about 5%). However, the interquartile range for the in vivo tests is much larger (as a proportion of the median) making discrimination more difficult as a stand-alone measure. As for the static modulus, increasing contact pressure or pre-strain increases the magnitude of the dynamic modulus for both in vivo and ex vivo measurements. The most likely reason for relatively low values of dynamic modulus in vivo is most probably because of the contact pressure being controlled manually and the natural reaction of the user to accommodate for the vibrations. This is further borne out by the fact that many of dynamic modulus measurements made at 1 Hz were unusable, hence they are not reported; these were amongst the earliest measurements made in vivo by this operator and were also at a lower frequency where accommodation is physiologically easier.

**Table 3.** Statistical indicators of dynamic modulus measured in vivo and ex vivo. Values quoted as median (interquartile range).

Patients	Pressure/Depth	Freq(Hz)	*E_dynamic_*C (kPa)	*E_dynamic_* NC (kPa)
*In vivo*				
15–22	L	5	3.12 (3.97)	2.81 (3.42)
15–22	F	5	5.15 (4.86)	4.61 (3.08)
*Ex vivo*				
15–22	3 mm	5	14.2 (0.90)	13.8 (0.54)
15–22	5 mm	5	15.1 (1.07)	14.3 (0.92)
15–22	8 mm	5	15.3 (0.21)	14.5 (1.86)

F: firm pressure applied; L: light pressure applied; C: point assessed as cancer; NC: point assessed as not cancer.

A basic principle of DIP (Figure 1) is that the phase lag between the force and the displacement will reveal the temporal response of the tissue, provided that the frequency of palpation is not too high. For practical application, there is a balance to be achieved in selecting the highest frequency which will yield diagnostically significant information in the shortest possible intervention time. From prior experience, the focus here is on 1 and 5 Hz frequencies which can reasonably be expected to allow a full examination within a few minutes’ intervention. Table 4 shows the statistical results for time lags for in vivo and ex vivo testing for the in vivo measurements and ex vivo measurements. For the in vivo measurements with firm pressure there is a small difference in time lags between the C and NC groups of the expected sense, that is that cancer is associated with shorter time constants in the tissue. However, this difference is smaller than the interquartile range and the results with light contact pressure have a much smaller difference, both as a percentage of the median and relative to the interquartile range. For the more controlled ex vivo conditions with the same patient group, the results are similar in that there is (with two exceptions) a small difference in time lags between the C and NC groups of the expected sense. However, the time lags for the ex vivo measurements are much larger than the in vivo and there is also a substantial drop in time lag between the smallest pre-strain and the other two. Finally, the time-lags measured at 1 Hz ex vivo were substantially larger than those measured at 5 Hz, which would suggest that the best frequency for testing lies below 1 Hz. Although the number of patients is relatively small, the 1 Hz in vivo results would seem to reinforce this interpretation.

**Table 4.** Statistical indicators of time lag between load and displacement measured in vivo and ex vivo. Values quoted as median (interquartile range).

Patients	Pressure/Depth	Freq(Hz)	*Δt* C (s)	*Δt* NC (s)
*In vivo*				
15–22	L	5	0.0178 (0.0048)	0.0173 (0.0062)
15–22	F	5	0.0175 (0.0078)	0.0186 (0.0397)
11–14	L	1	0.0204 (0.0261)	0.0261 (0.2514)
11–14	F	1	0.0281 (0.0500)	0.0422 (0.4549)
*Ex vivo*				
15–22	3 mm	5	0.0286 (0.0.0020)	0.0287 (0.0011)
15–22	5 mm	5	0.1430 (0.0140)	0.1430 (0.0110)
15–22	8 mm	5	0.1412 (0.0205)	0.1398 (0.0174)
15–22	3 mm	1	0.0418 (0.0039)	0.0420 (0.0029)
15–22	5 mm	1	0.4183 (0.0055)	0.4196 (0.0048)
15–22	8 mm	1	0.4167 (0.0075)	0.4201 (0.0075)

F: firm pressure applied; L: light pressure applied; C: point assessed as cancer; NC: point assessed as not cancer.

In order to complement the above statistical approach and point the way towards diagnostic indicators for DIP, a pattern recognition approach has been adopted using a proprietary Artificial Neural Network (ANN) algorithm (MATLAB). Here, all of the features (mean ratio, amplitude ratio, and phase and/or time difference at each of the frequencies and contact pressures/depths) for the ex vivo tests^22^ and the in vivo tests.

**Figure 5.**
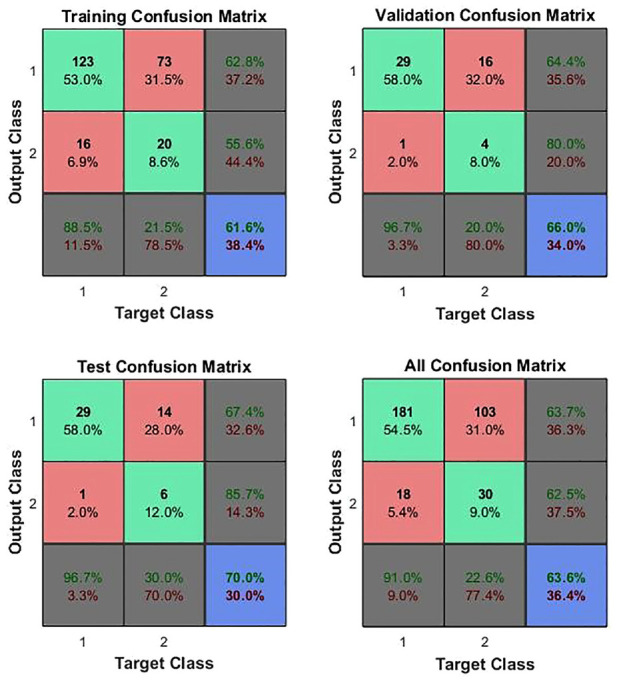
ANN confusion matrices for in vivo measurements on patients 11–22, assessed at either 1 or 5 Hz. Class 1 is C and Class 2 is NC and so element 1,1 represents true positives, 1,2, false positives, 2,1 false negatives and 2,2, true negatives.

**Figure 6.**
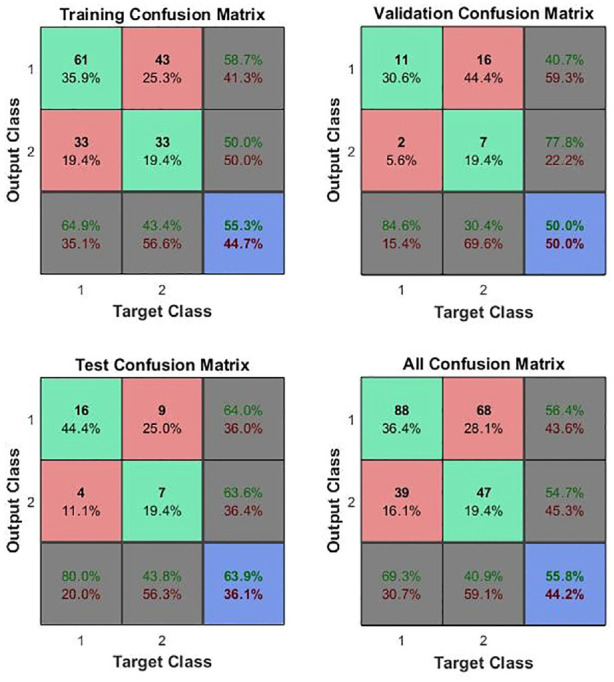
ANN confusion matrices for in vivo measurements on patients 15–22, assessed at 5 Hz. Class 1 is C and Class 2 is NC so element 1,1 represents true positives, 1,2, false positives, 2,1 false negatives and 2,2, true negatives.

The ANN was presented with a mechanical feature vector for each point probed, for all of the patients in each of the ex vivo and in vivo groups (232 points ex vivo and 165 points in vivo) and subjected to 10-fold cross validation using scaled conjugate back-propagation on a random selection of 70% of the data as described in Section 2.3. The validation and test runs were each carried out on a random selection of 15% of the data. The network confusion matrix for the 232 × 18 mechanical feature vector for the ex vivo tests has been published elsewhere.^22^Figures 5 and 6 show those for the whole in vivo patient group^11–22^ and for those in vivo patients^15–22^ on whom a 5 Hz measurement was made. In both cases, the mechanical vector consisted of eight features, *E_static_*, *E_dynamic_*, phase difference and *Δt*, at each of firm and light pressure. It might be noted that each patient was only assessed at a single frequency, so the in vivo feature vector is less complete than that for the ex vivo measurements and is not quite congruent across the patient set.

Table 5 summarises the diagnostic capabilities indicated by the test confusion matrices for the ex vivo assessment and for the two in vivo ANN assessments. For the entire patient group (first two rows), all of the indicators are considerably improved in vivo, despite the impoverished feature matrix for the in vivo measurements and the fact that the registration of the in vivo tests against the pathology sections is not as precise as the ex vivo tests. Comparing the single-frequency in vivo group with the full group, there is a clear improvement in specificity, but a reduction in sensitivity, indicating that a more focused feature set will overcome the relatively poor specificity.

**Table 5.** Diagnostic capability summary for the in vivo and ex vivo^22^ patient groups.

Method	Number of pointsassessed	Diagnostic indicator (%)				
		Sensitivity	Specificity	NPV	PPV	Accuracy
Ex vivo DIP^22^	232	93	16	59	64	64
In vivo DIP 1/5 Hz	165	97	30	86	67	70
In vivo DIP 5 Hz	120	80	44	64	64	64

## Discussion

One of the main purposes of the present paper is to contribute to knowledge on the mechanical properties of prostate tissue and on the effect of cancer. The issue has been of interest since the early 1990s, initially for ultrasonics applications,^23^ and more recently for probes using direct mechanical assessment.^22,24^ Relatively few direct mechanical studies have used whole prostate samples,^22,25,26^ few have measured viscoelastic properties^17,22^ and none, until now, have made measurements in vivo.

Table 6 summarises all reported static modulus measurements on whole prostates, including the current work and the corresponding ex vivo measurements.^22^ Of the other two sets, the measurements of Ahn and Kim^26^ most closely correspond in terms of loading rate and classification. As can be seen, the current in vivo measurements are reasonably close to those of Ahn and Kim with a similar spread in measurement, but with a smaller discrimination between mean (or median) C and NC classifications. In both cases, the variability is much smaller than the work of Carson et al. who have not considered NC tissue and focus on palpability. The ex vivo measurements of the current authors stand out as having low modulus, little discrimination between C and NC and a very small variability. It is not clear why this is, but may be associated with the overlapping measurements and the classification of C and NC in terms of the histological section. This area will require some further research including the use of a model to relate histology to palpability.

**Table 6.** Literature values of quasi-static modulus measured on whole prostates. Values quoted for current work as median (interquartile range).

Reference	Methodology	*E_static_* C (kPa)	*E_static_* NC (kPa)
22 Current work (ex vivo)	11 patients, sinusoidal indentation at up to 35 points per patient, 1 and 5 Hz, pre-strains of 3, 5 and 8 mm, classified as any cancer in column below indenter.	14.9 (0.75)	14.8 (0.77)
25 (Carson et al.)	32 patients, unload indentation from 9 mm over around 10 min, 5 points per patient, classified as palpable vs non-palpable abnormalities	46.5 ± 22.2	31.0 ± 63.1
26 (Ahn and Kim)	35 patients, indentation from 0 to 3 mm at 1 mm/s at 21 points per patient, classified as cancerous and normal regions.	28.8 ± 11.2	15.2 ± 5.8
Current work (in vivo)	12 patients, sinusoidal indentation at up to 16 points per patient in vivo, 1 and 5 Hz, firm and light manual pressure, classified as any cancer in column below indenter.	26.8 (13.3)	24.8 (11.9)

C: assessed as cancer; NC: assessed as not cancer.

Table 7 summarises all reported dynamic mechanical measurements on prostate and bladder tissue where cancer is a focus. No other authors have measured dynamic mechanical properties on whole prostates, although Zhang et al.^17^ have measured time-dependent behaviour on 17 prostate cores from eight patients, using a stress relaxation test at 5% strain over a period of 700 s. They fitted their results to a Kelvin-Voigt viscoelastic model and extrapolated to a dynamic modulus at 150 Hz to find the storage and loss modulus. The same model can be used to extrapolate (with more justification) to find the storage and loss modulus at 1 and 5 Hz. The only other (reasonably) comparable study is by Barnes et al.^27^ who measured storage modulus (E′) and loss modulus (E″) for seven samples of bladder cancer tumours, fitting curves of each modulus as a function of frequency of testing over the range 0.01 to 50 Hz. The two component moduli (along with those of Zhang et al.) have been converted to time lags and dynamic moduli in Table 7, to facilitate comparison with the current work, both in vivo and ex vivo. Overall, there is general agreement that the dynamic modulus for C classifications is higher than for NC, and, apart from the ex vivo tests of the current authors,^22^ there is remarkable agreement on the magnitude of the time differences and the sense of change with frequency and between C and NC conditions.

**Table 7.** Comparison of reported dynamic mechanical measurements of human prostate and bladder tissue for C and NC conditions. (Data from current study includes only firm contact and 8 mm pre-strain).

Reference	*n*	Freq. (Hz)	*Δt* C (s)	*Δt* NC (s)	*E_dynamic_* C (kPa)	*E_dynamic_* NC (kPa)
Current work (in vivo)	4	1	0.0281 (0.0500)	0.0422 (0.4549)		
Current work (in vivo)	8	5	0.0175 (0.0078)	0.0186 (0.0397)	5.15 (4.86)	4.61 (3.08)
Barnes et al.^27^	7	1	0.038		71	
Barnes et al.^27^	7	5	0.009		79	
Zhang et al.^17^	17	1	0.056 ± 0.007	0.054 ± 0.010	13.07 ± 5.88	5.36 ± 2.11
Zhang et al.^17^	17	5	0.011 ± 0.002	0.011 ± 0.002	18.76 ± 9.39	7.59 ± 3.75
Hammer et al.^22^	12	1	0.4167 (0.0075)	0.4201 (0.0075)		
Hammer et al.^22^	12	5	0.1412 (0.0205)	0.1398 (0.0174)	15.3 (0.21)	14.5 (1.86)

C: assessed as cancer; NC: assessed as not cancer.

**Figure 7.**
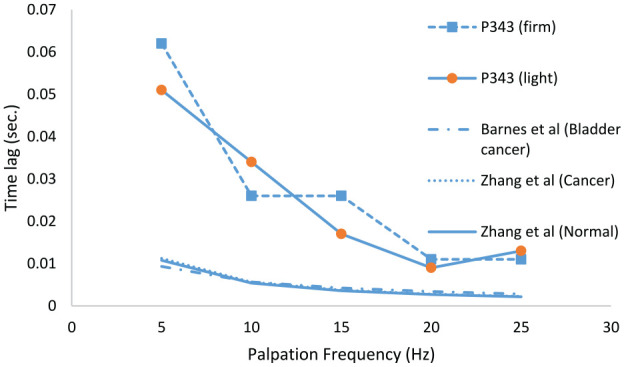
Evolution of measured, non-indexed in vivo time lags from Patient 343 over five actuation frequencies compared with ex vivo-based models of Zhang et al.^17^ and Barnes et al.^27^

As mentioned in section 2.1, one set of data (Patient no. 343) from a larger (and later) as-yet unpublished study by the current authors is included here as it involved a wider sweep of frequencies than the current study. Figure 7 shows the measured time lags with firm and light pressure, averaged over the 12 probe points as a function of frequency and compared with the (ex vivo-based) models of Zhang et al.^17^ for C and NC prostate and of Barnes et al.^27^ for bladder cancer. Given that the Patient 343 was about to undergo radical prostatectomy for cancer, it is likely that 50% or more of the points probed will be of C classification. The magnitude of the time lag for the in vivo test on P343 is about five times what it is for the two ex vivo tests, an observation which can be explained by the latter being small, excised samples with limited capacity to store expressed water from under the indenter. However, it is interesting to note that all the curves show a similar type of evolution with frequency which indicates that the dynamic probing method is a good way of assessing the time-based (i.e. viscous) behaviour of the tissue yielding a measure (time) which does not require calibration. However, taken with the statistical indicators in Table 7, it is clear that a wider data set of frequencies is needed to exploit the diagnostic capacity of time-based mechanical probing to its maximum potential. As well as these shorter-time effects (which are really only suitable for elastography), the data contain some longer-time behaviour measurable as a drift in the mean over the period of testing. At present, these drifts are extracted from the data, but future studies will focus on obtaining longer timescale observations.

Li et al.^15^ have compared the diagnostic capabilities of their rolling mechanical indenter (RMI) measurements with a number of other diagnostic methods on 22 patients who had undergone radical prostatectomy. The RMI was scanned over the surface of the prostate at an average speed of 10 mm/s at a depth of 3 mm, recording force using a proprietary 6-DOF force/torque sensor. They used five indicators:

Sensitivity = $\%\left(\frac{\mathrm{TP}}{\mathrm{TP}+\mathrm{FN}}\right)$ , Specificity = $\%\left(\frac{\mathrm{TN}}{\mathrm{TN}+\mathrm{FP}}\right)$ , Negative predictive value (NPV) = $\%\left(\frac{\mathrm{TN}}{\mathrm{TN}+\mathrm{FN}}\right)$

Positive predictive value (PPV) = $\%\left(\frac{\mathrm{TP}}{\mathrm{TP}+\mathrm{FP}}\right)\;$ and Accuracy = $\%\left(\frac{\mathrm{TP}+\mathrm{TN}}{\mathrm{TP}+\mathrm{FP}+\mathrm{FN}+\mathrm{TN}}\right)$

where ‘T’ and ‘F’ and ‘P’ and ‘N’ have the usual logical meanings (e.g. TP = True positive).

Table 8 shows the results of these RMI tests compared with the conventional methods (DRE, MRI and TRUS) alongside the diagnostic capabilities indicated by the test confusion matrices for the ex vivo assessment and for the two in vivo ANN assessments (Table 5). As can be seen, the sensitivity of the current method is better than RMI, DRE, MRI and TRUS, as evaluated by Li et al, although the specificity is poorer across the board. For a screening test, however, the most important diagnostic indicator is negative predictive value, and the full in vivo data set shows an NPV considerably higher than RMI, DRE, MRI or TRUS. It is worth pointing out that the statistical comparison with the work of Li et al. is not like-for-like since theirs is based on a single parameter *t*-test, whereas the ANN effectively samples the distributions of a range of features.

**Table 8.** Diagnostic capability summary for in vivo and ex vivo assessments of the current patient group (Table 5), compared with the ex vivo study of RMI by Li et al.^15^

Method	Number of pointsassessed	Diagnostic indicator (%)				
		Sensitivity	Specificity	NPV	PPV	Accuracy
Ex vivo DIP^22^	232	93	16	59	64	64
In vivo DIP 1/5 Hz	165	97	30	86	67	70
In vivo DIP 5 Hz	120	80	44	64	64	64
RMI^15^	126	44	71	56	61	58
DRE^15^	126	38	67	52	53	52
MRI^15^	126	33	82	55	64	58
TRUS^15^	126	76	52	69	62	64

In parallel work,^28^ an inverse finite element approach has been developed to improve the precision of interpretation of the point probing results. Assuming the C and NC areas to be homogeneous with static moduli *E_C_* and *E_NC_*, the reaction force profile can be simulated using inverse FEA methods so that the difference between reaction force profiles from the FE model and the experimental measurements is minimised. For an effective probe diameter the same as for the current in vivo tests, values of the two best fit moduli become less distinct as the probe depth diminishes and the fit of the simulated profile to the measured one deteriorates. The performance, and the detectability of a given nodule or series of nodules depends on a number of assumptions about the boundary conditions, probe size and probe depth. Although this approach has not yet been rigorously applied in parallel with in vivo measurements, it is clear that adopting a ‘mechanically intelligent’ probe where the moduli can first be measured in vivo and, later, the measurements can be used to locate the size and depth of any tumours is extremely powerful, not only for the current application, but for minimally invasive tissue identification generally. Preliminary validation using silicone phantoms^29^ and incorporating time-dependent response^18^ are promising.

## Conclusions

One advantage of the current in vivo probe is that it returns values that can be traced to measurement standards and so allows a modulus to be determined in kPa. The work has established the first measurements of the static modulus of living prostate tissue to be 26.8 (13.3) kPa for tissue affected by prostate cancer (C classification) and 24.8 (11.9) for tissue unaffected by cancer (NC classification), values quoted as median (interquartile range). In both cases the condition is attributed to a column of tissue immediately underneath the indenter and, for cancer, much of the variation is expected to be the result of the size and depth of any lesions. For the NC classification, the variation may be a result of the individual patient variability and/or spatial variability in the anatomy of the prostate and/or other disease conditions, such as BPH.

Another advantage of the current in vivo probe is that it takes account of viscous as well as elastic effects, measuring these as a difference in phase between load and displacement, which can then be converted to a time difference, comparable with relaxation times measured in conventional stress relaxation or creep experiments. The work has established the first measurements of relaxation times in living prostate tissue at 5 Hz palpation frequency of 0.0175 s (0.0078) for the C classification and 0.0186 s (0.0397) for the NC classification, values again quoted as median (interquartile range). The variation is likely to be attributable to the factors mentioned above, as well as the fact that time lags are clearly a function of frequency of palpation.

The dynamic moduli were only measured with sufficient sensitivity at 5 Hz yielding values of, 5.15 kPa (4.86) for the C classification and 4.61 kPa (3.08) for the NC classification. These values compared reasonably well with the nearest equivalent ex vivo measurements from the literature, with the exception of the ex vivo work of the current authors.^22^ A more complete set of measurements over a wider range of frequency is expected to yield a characteristic change of time lag and dynamic modulus with frequency which will aid cancer identification with greater resolution.

The current modulus measurements assume a Hertzian stress distribution below the indenter, irrespective of its homogeneity. Future work will include the introduction of FE-based ‘mechanical intelligence’ into the extraction of the moduli of the cancer and the matrix material and so improve the distinction between C and NC and also introduce a severity level for the C classification.

The current embodiment of the probe includes a hold-down force sensor which was designed only to detect contact with the sample surface for the displacement-controlled ex vivo tests.^22^ The probe design will need some development to incorporate a more sensitive hold-down force sensor to allow better measurement (and some control) of the contact force during clinical application. This is expected to yield further features which can be used to improve the diagnostic capability of the probe.
